# Advances and Potentials of Polydopamine Nanosystem in Photothermal-Based Antibacterial Infection Therapies

**DOI:** 10.3389/fphar.2022.829712

**Published:** 2022-03-07

**Authors:** Shuhao Fan, Wensen Lin, Yifan Huang, Jiaojiao Xia, Jun-Fa Xu, Junai Zhang, Jiang Pi

**Affiliations:** Institute of Laboratory Medicine, Guangdong Provincial Key Laboratory of Medical Molecular Diagnostics, School of Medical Technology, The First Dongguan Affiliated Hospital, Guangdong Medical University, Dongguan, China

**Keywords:** photothermal therapy, bacterial infections, anti-bacterial therapy, polydopamine nanosystem, biological functions and mechanisms

## Abstract

Bacterial infection remains one of the most dangerous threats to human health due to the increasing cases of bacterial resistance, which is caused by the extensive use of current antibiotics. Photothermal therapy (PTT) is similar to photodynamic therapy (PDT), but PTT can generate heat energy under the excitation of light of specific wavelength, resulting in overheating and damage to target cells or sites. Polydopamine (PDA) has been proved to show plenty of advantages, such as simple preparation, good photothermal conversion effects, high biocompatibility, and easy functionalization and adhesion. Taking these advantages, dopamine is widely used to synthesize the PDA nanosystem with excellent photothermal effects, good biocompatibility, and high drug loading ability, which therefore play more and more important roles for anticancer and antibacterial treatment. PDA nanosystem-mediated PTT has been reported to induce significant tumor inhibition, as well as bacterial killings due to PTT-induced hyperthermia. Moreover, combined with other cancer or bacterial inhibition strategies, PDA nanosystem-mediated PTT can achieve more effective tumor and bacterial inhibitions. In this review, we summarized the progress of preparation methods for the PDA nanosystem, followed by advances of their biological functions and mechanisms for PTT uses, especially in the field of antibacterial treatments. We also provided advances on how to combine PDA nanosystem-mediated PTT with other antibacterial methods for synergistic bacterial killings. Moreover, we further provide some prospects of PDA nanosystem-mediated PTT against intracellular bacteria, which might be helpful to facilitate their future research progress for antibacterial therapy.

## Introduction

Bacterial infections continue to represent a primary worldwide health issue (McEwen and Collignon, 2018; Brink, 2019). In recent years, with the overuse of antibiotics, bacterial drug resistance has become a more and more challenging issue due to the emergence of multi-resistant mutants and the limited antibiotics for effective control of drug resistance. Some deadly drug-resistant bacteria, such as drug-resistant *Mycobacterium tuberculosis* (*M. tuberculosis*, Mtb) and drug-resistant *Staphylococcus aureus* (*S. aureus*), are causing millions of deaths every year worldwide (Rendon et al., 2017; Lee et al., 2018). Thus, it is of vital importance to develop novel therapeutics against bacteria and drug-resistant bacterial infections.

Photodynamic therapy (PDT) is a noninvasive therapy strategy that has been developed for the treatment of dental caries (Nagata et al., 2012), wound infection (Lee et al., 2018; Yang H. et al., 2021), and various sites and types of cancer (Allison et al., 2011; Azzouzi et al., 2017). After light irradiation with a specific wavelength, the photosensitive compounds, also called photosensitizers, would be activated and energy transferred to produce singlet oxygen (^1^O_2_) and other reactive oxygen species (ROS), which can selectively induce cell apoptosis and microbial inactivation (Allison et al., 2011; Chen et al., 2016). Based on the local or systemic application of photosensitizers, PDT has been proved by the Food and Drug Administration (FDA, USA), as well as some other countries, for anticancer treatment against some specific cancers (Dabrowski and Arnaut, 2015). Similar with the PDT strategy, photothermal therapy (PTT) has recently been developed based on photothermal conversion materials to convert light energy into heat under light excitation of specific wavelength, resulting in excessive heat and leading to protein denaturation or damage to bacteria and cancer cells (Yang et al., 2018). With the development of nanotechnology, the combined application of functional nanomaterials and PTT has introduced novel possibilities for the development of more effective anticancer and antibacterial treatments (Wang et al., 2019a; Li et al., 2019; Zhang et al., 2020).

Dopamine (DA) is the most abundant catecholamine neurotransmitter in the brain, which regulates various physiological functions of the central nervous system as a neurotransmitter (Pinoli et al., 2017). Dysregulation of the DA system has been found to be involved in some very important diseases, such as Parkinson’s disease, schizophrenia, attention deficit hyperactivity syndrome, and pituitary tumor (Klein et al., 2019). Recently, more and more researchers have found that polydopamine (PDA) has the advantages of simple preparation, high biocompatibility, easy functionalization, and good adhesion, which therefore show strong potentials to act as a drug carrier for targeted delivery after the modification with different ligands (Li H. et al., 2021). PDA nanomaterials have been proved to show low cytotoxicity, low residual biological activity, and specific binding ability to target cells (Park et al., 2014; Su et al., 2020), which suggested their promising future for targeted therapy. Additionally, PDA can also be used for the preparation of residual sensors to significantly improve their sensitivity and stability (Yao et al., 2020).

More interestingly, the coating of some nanomaterials with PDA could introduce some advanced characteristics for the nanosystem, such as low cytotoxicity, high biocompatibility, strong near-infrared absorption, good thermal stability, and high photothermal conversion efficiency (Singh et al., 2020; Wu et al., 2021). Taking the advantages of high photothermal conversion efficiency, PDA nanomaterials have been widely applied for anticancer treatment, which can directly inhibit cancer cell growth by promoting autophagy, apoptosis, and so on (Hao et al., 2019; Wu et al., 2020; Dai et al., 2021a). Moreover, combining targeted drug delivery and PTT, the PDA nanosystem is expected to synergistically eliminate tumor growth (Wu et al., 2020; Dai et al., 2021a; Dai et al., 2021b). Notably, PDA nanomaterial and PTT combined therapy can also enhance anticancer immunity that contributes to tumor inhibition, indicating the promising potential of PTT for more advanced anticancer strategy development (Liu et al., 2021).

Inspired by the PTT-based anticancer strategy, PTT has also been explored in different bacterial systems to explore their potential for antibacterial treatments (Xu M. et al., 2020; Zhao et al., 2020). The light-transferred heat could not only directly destroy bacterial structure and functions (Yuwen et al., 2018) but also introduce more potent antibacterial immunity against intracellular bacterial infections (Yan M. et al., 2019). Moreover, due to the low cytotoxicity and high biocompatibility of PDA, PDA nanomaterials can be widely used for antibacterial treatment with few side effects (Hu et al., 2017). Herein, in this review, we will summarize the recent exciting progress of PDA nanomaterials for antibacterial PTT treatment, which might benefit future antibacterial strategy developments.

## Preparation and Polymerization Mechanism of PDA Nanosystem

PDA is a kind of adhesive polymer that can functionalize different surfaces made of virtually all material chemistries by DA polymerization. The exact mechanism of DA polymerization and its structure have not yet been clearly explained, which remains to be further explored. Hong et al. reported a physical, self-assembled trimer of (dopamine)_2_/5,6-dihydroxyindole that exists in PDA, which was proved to be formed only by covalent polymerization (Hong et al., 2012). This study revealed a perspective of PDA formation, where it forms in part by the self-assembly of DA. Under alkaline conditions, DA is oxidized to generate DA quinone, and after nucleophilic reaction and oxidation reaction, 5,6-dihydroxyindole is finally formed. 5,6-Dihydroxyindole can form PDA through the following two typical pathways. The first pathway is that 5,6-dihydroxyindole can form a (dopamine)_2_/5,6-dihydroxyindole trimer with unpolymerized DA through its own covalent bond. The second pathway is that the (dopamine)_2_/5,6-dihydroxyindole trimer is tightly attached to PDA through non-covalent interactions such as T-shaped interactions, H-bonds, cationic-π interactions, and oxidative polymerization products (Hong et al., 2012; Liu et al., 2014).

**FIGURE 1:**
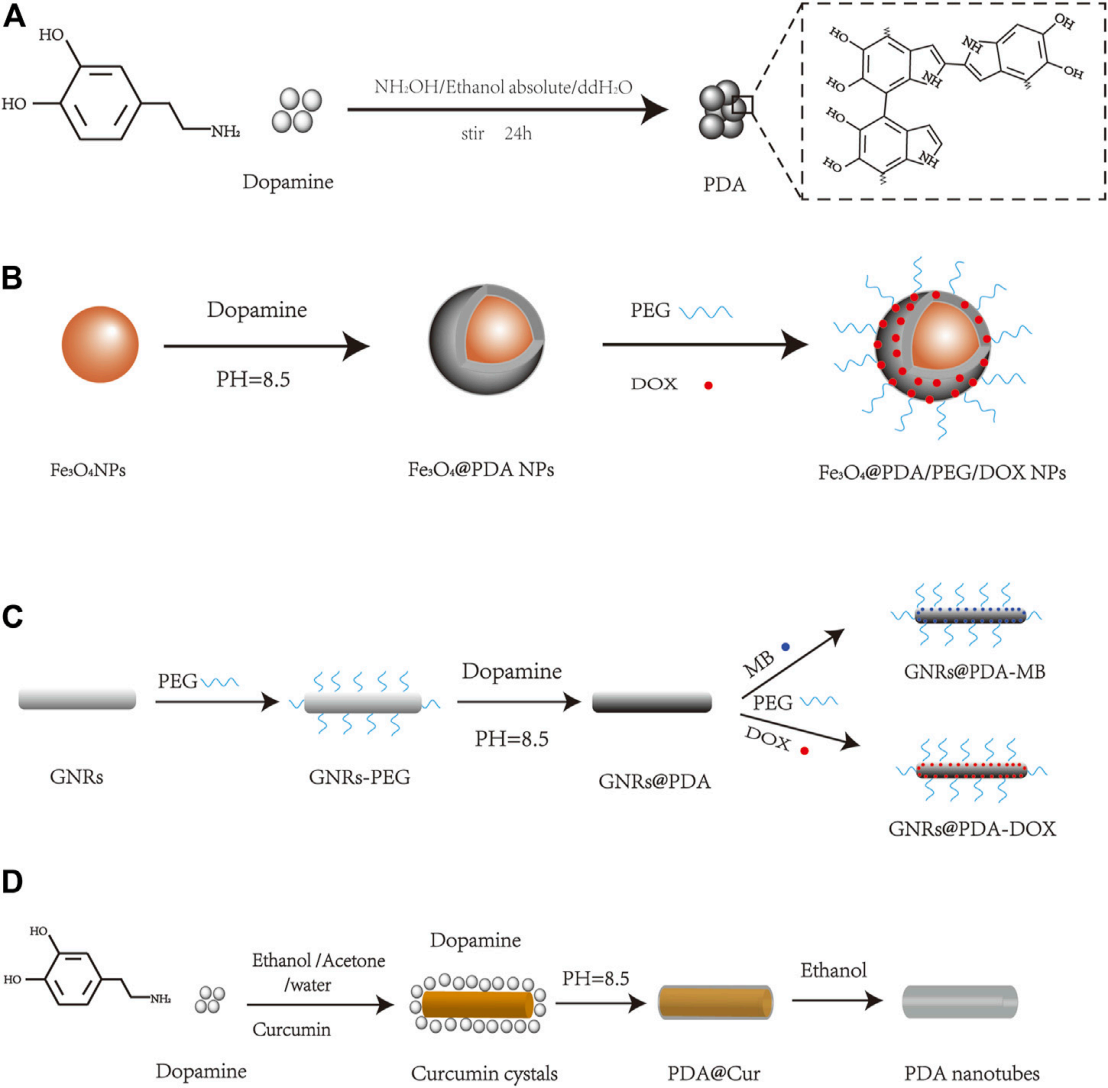
Schematic diagram of PDA nanomaterial synthesis, such as **(A)** PDA NPs (Liu et al., 2013), **(B)** Fe_3_O_4_@PDA/PEG/DOX NPs (Xue et al., 2017), **(C)** GNRs@PDA (Wang S. et al., 2016), and **(D)** PDA nanotubes (Xue et al., 2016).

There are some well-established PDA synthesis methods, such as enzyme oxidation, electropolymerization, and solution oxidation. Due to the simple operation and convenient synthesis, solution oxidation has become the most widely used method to prepare PDA. Currently, there are two common solution oxidation synthesis methods: 1) to prepare PDA nanoparticles, dopamine hydrochloride is generally added to the mixed solution of ammonia/anhydrous ethanol/deionized water, stirred at room temperature, then centrifuged to collect PDA nanoparticles (Figure 1A) (Liu et al., 2013); 2) PDA-coated nanoparticles were prepared by adding DA to the precursor of the target nanoparticles in Tris–HCl buffer solution at the designed pH and stirred, and the surface of the nanomaterials was coated with PDA by self-polymerization of DA (Liu et al., 2013; Liu et al., 2014; Yang et al., 2017). The second method provides the convenience to synthesize PDA nanomaterials combining some other nanomaterials inside with different shapes, which will be discussed later.

There are many factors affecting DA polymerization in the solution oxidation process. Firstly, the buffer and solvent directly affect PDA polymerization. A study shows that different buffer solutions (Tris and PBS) have different impacts on PDA particle size, which may be attributed to the nucleophiles in Tris buffer that can be targeted to regulate particle formation, resulting in a much smaller PDA particle size formation in Tris buffer than in PBS (Della Vecchia et al., 2014). Moreover, the size of the PDA nanoparticles can also be adjusted by changing the ratio of the water and ethanol mixture solution (Yan et al., 2013).

Secondly, the oxidation kinetics of DA show that pH, temperature, oxidant, and DA concentration would affect the main process of DA polymerization by affecting the oxidation rate. Many studies have demonstrated that self-polymerization of DA occurs at pH = 8.5. Bernsmann et al. found that the thickness of the PDA film could reach more than 70 nm with the addition of oxidant, while the thickness of the PDA film was only about 45 nm without the addition of oxidant, and the self-polymerization of DA could not occur at a pH less than 7 (Bernsmann et al., 2011). The thickness and roughness of the film always increase with the increased DA concentration (Ball et al., 2012), and the rate of DA polymerization would also increase with the increased reaction temperature (Zhou et al., 2014).

Finally, other influencing factors, such as substrate and external stimulus, can also affect DA polymerization. In the study by Zhou et al., it was proved that the polymerization of DA could be accelerated not only by temperature but also by agitation (Zhou et al., 2014). Lv et al. used PDA/polyethyleneimine (PEI) co-deposition experiments to verify the relationship between PDA adsorption rate and substrate material (Lv et al., 2018). Additionally, some factors such as UV light exposure and reaction time were also found to affect DA polymerization (Du et al., 2014). Thus, it is of vital importance to control the reaction conditions in the preparation of PDA nanomaterials.

Due to its easy polymerization and adhesive property, PDA can be combined with different nanomaterials to form different nanostructures. At present, PDA-based nanomaterials with different shapes have been reported, such as spherical, rod, tube, or other shapes. Spherical PDA nanomaterials are the most common forms, such as Fe_3_O_4_@PDA NPs and MSN-Man-DOX@PDA (Xue et al., 2017;Li et al., 2021c). For example, the paramagnetic Fe_3_O_4_ can be combined with PDA to form spherical nanoparticles for tumor drug delivery, which can be easily collected and purified (Figure 1B) (Xue et al., 2017). A simple and versatile rod-shaped GNR@PDA NP nanoplatform that combines DOX and MB-mediated photodynamic/photothermal therapy and chemotherapy was also reported to achieve significant high anticancer efficacy (Figure 1C) (Wang S. et al., 2016). Xue et al. synthesized tube-like particles combining curcumin crystals and PDA, and the purified PDA nanotubes with microporous and mesoporous characteristics showed potential to load curcumin and improve sensor sensitivity (Figure 1D) (Xue et al., 2016). In addition to the abovementioned materials, star, sheet, capsule, and other shapes of PDA nanomaterials can also be obtained (Hu et al., 2016; Li et al., 2016; Sun et al., 2018; Liu et al., 2019), providing potential to prepare different functional PDA nanomaterials for biomedical use.

## Functions of PDA Nanosystem Mediated PTT

With the development of nanotechnology, nanomaterial-based systems can accumulate into the tumor site through enhanced permeability and retention (EPR) effect and be further enhanced by the functional modification with specific ligands (Kang et al., 2020; Yu et al., 2020). Recently, PDA nanomaterials have been reported to show very good biological characteristics, such as low cytotoxicity, excellent biocompatibility, and high photothermal efficiency (Wang X. et al., 2016; Wang et al., 2017; Zhang et al., 2017). The photothermal effects of nanomaterials, a kind of ability to convert light irradiation with a certain wavelength into heat, have been highlighted as a kind of novel antitumor strategy by specifically increasing the temperature of tumor. A lot of studies have reported that the PDA nanosystem has very good photothermal effects for PTT, and the high heat generated by the PDA nanosystem after irradiation can effectively destroy tumor cells. Therefore, PDA nanomaterials, when combined with PTT, can be applied with great potential for antitumor treatment (Wang X. et al., 2016; Zhang et al., 2017; Yu et al., 2020).

Taking the advantages of the high drug encapsulation efficiency of the PDA nanosystem, drug delivery can also be introduced to combine with PTT for synergetic antitumor treatment (Wu et al., 2018; Chen et al., 2020). Fan et al. compounded Fe_3_O_4_@PDA-PEG-cRGD-DOX NPs to deliver DOX to tumor cells, and the release of DOX was regulated by photothermal and pH conditions to achieve maximum therapeutic effects (Fan X. et al., 2019). The research of Zhang et al. also demonstrated that the PDA nanosystem had excellent drug delivery and photothermal effects for enhanced antitumor efficiency (Zhang T. et al., 2019).

Due to the high encapsulation efficiency and controllable drug release ability, the PDA nanosystem therefore shows broad application potentials by combining chemotherapy and PTT for antitumor treatment (Wang et al., 2018). The insufficient heat damage induced by PTT cannot always completely eradicate tumor cells due to the presence of multiple intracellular defense mechanisms in cancer cells, especially “autophagy,” which is activated by cancer cells to avert and repair cellular damage caused by PTT (Amaravadi et al., 2019; Zhang et al., 2022). Autophagy is a self-degradation process that maintains cellular homeostasis through degradation of misfolded proteins or damaged organelles by the lysosomal system (Choi et al., 2013). It has been proved that autophagy plays very vital roles in tumor formation and progression, which shows that autophagy can serve as a pro-survival mechanism in cancer cells against multiple therapeutic stresses by accelerating the self-repair of damaged cells and weakening the killing effects of anticancer therapies (Folkerts et al., 2019). Therefore, the synergistic modulation of autophagy that augments cancer cell sensitivity to diverse therapies is proposed as a potential strategy for more effective cancer therapy (Zhou et al., 2017). Heat generated from PTT can damage the cytoplasmic components of cancer cells, which can promote the cancer cells to activate protective autophagy to resist PTT therapeutic stress (Zhang and Calderwood, 2011; Ahmed et al., 2020). Thus, there is an emerging strategy to enhance anticancer efficiency of PTT by blocking/inhibiting the protective autophagy of cancer cells. Moreover, based on this strategy, we previously fabricated porous PDA nanoparticles to encapsulate autophagy inhibitor chloroquine (CQ) inside, which resulted in much stronger tumor inhibition combined with PDA nanoparticle-mediated PTT (Huang et al., 2021). Similar results were also obtained by using autophagy inhibitor CQ to inhibit PTT-induced protective autophagy in cancer cells, which led to enhanced tumor inhibition/killing efficiency (Wang X. et al., 2020; Shao et al., 2020).

Interestingly, some works have proved that inhibition of autophagy can also promote apoptosis to enhance the killing effect on tumor cells (Wang et al., 2019b; Wang Y. et al., 2019). Apoptosis is a form of programmed cell death and can be activated by compounds targeting intrinsic and extrinsic pathways, which is therefore widely used as targets for cancer therapy by some current anticancer drugs. It has been recently reported that tissues treated by the PDA nanosystem will promote apoptosis signaling events after irradiation (Liu et al., 2020; Luo et al., 2020). Moreover, the mechanism has been proved that the PDA nanosystem can induce both caspase-3 dependent and non-dependent apoptosis to kill tumor cells in experiments (Hao et al., 2019). Zhao et al. also found that the PDA nanosystem could increase the expression of Bax and decrease the expression of bcl-2, which resulted in mitochondrial membrane potential damage and the release of activated cytopigment C to activate the caspase-3/caspase-8 apoptosis pathway (Zhao et al., 2018).

In addition to the abovementioned cancer cell death mechanisms, the PDA nanosystem has also been found to activate antitumor immunity. For example, Fe@PDA-PEG could not only mediate PTT for tumor killing but also repolarize M2 tumor-associated macrophages to M1 macrophages as primary phagocytes, which significantly activate antitumor immunity (Rong et al., 2019). Furthermore, the PDA nanosystem could also promote dendritic cell maturation and activate CD8+T lymphocytes to enhance antitumor immunity (Yan S. et al., 2019; Wang D. et al., 2020). Such immunological regulation effects of the PDA nanosystem provide novel possibilities to develop antitumor immunotherapy strategy based on the PDA nanosystem.

Generally, the PDA nanosystem can introduce hyperthermia in tumor by PTT, which can be combined with other strategies, including autophagy inhibition, apoptosis induction, and immune regulation, to achieve synergistically enhanced antitumor efficiency. These advantages of the PDA nanosystem provide more ideas to apply PTT for more infective antitumor treatment, which also introduces new insights for the development of antibacterial strategies.

## Anti-Bacterial Effects of PTT Mediated by PDA Nanosystem

Nanomaterial-based systems with high photothermal efficiency can be approved for PTT, which has been found to show broad spectra of antimicrobial activities with no bacterial resistance compared to some traditional antimicrobial methods (Wang et al., 2021). Photosensitizers play a central role in the bactericidal efficacy of PTT. Therefore, the development of powerful nanosystems with high photothermal efficiency has attracted great attention and interest.

Currently, there are five types of nanoscale photosensitizer commonly used, which contain noble metal nanomaterials, mental sulfide/oxide nanomaterials, carbon-based nanomaterials, polymer-based nanomaterials, and small molecule-based nanomaterials (Yougbare et al., 2021). The antibacterial function of these nanosystems is based on their accumulation in bacteria by targeting effects, and the irradiation will introduce irreversible heat killings such as cell membrane damage and protein denaturation in bacteria (Cui et al., 2020). PTT therapy alone often fails to kill bacteria completely; however, the combination of some antibacterial drugs with nanomaterial-mediated PTT could significantly enhance the antibacterial effects of nanosystems (Cui et al., 2020). This PTT and drug synergetic killing strategy is developed from the well-established synergetic antitumor PTT strategy, which is expected to develop more potent antibacterial techniques.

### Direct Bacteria Killing Effects of PDA nanosystem Mediated PTT

Different nanomaterials always show different antibacterial properties; for instance, PDA-coated gold nanorods could serve as a practical platform for use in chemothermal focal infection therapy, mental sulfide/oxide nanomaterials could disrupt the oxidation balance of bacteria, and carbon-based nanoparticles could affect the oxidative stress and bacterial encapsulation (Liu et al., 2018; Wang X. et al., 2019; Seifi and Kamali, 2021). Therefore, the main antibacterial mechanism of the PDA nanosystem for antibacterial PTT treatment is always dependent on the internal materials inside the PDA nanosystem. The PDA nanosystem mainly relies on photothermal effects to destroy the normal structure of bacteria to kill bacteria. Moreover, the photothermal efficiency of PDA nanomaterials not only is related to the size, shape, and charge of the nanomaterials but also can be improved by optimizing the laser parameters to improve the effect of nanomaterial-mediated PTT. At present, the main strategies focus on the optimization of wavelength, power density, radiation location, radiation number, and radiation intensity to improve the photothermal efficiency of PDA (de Melo-Diogo et al., 2017). PDA nanosystems have been proved to have good photothermal performance and stability and are still commonly used for treatment according to the international standard of 808 nm. However, due to the uneven heat distribution of the PDA nanosystem on bacteria and the potential side effects of high temperature on normal cells nearby, this method is still under investigation for its feasibility in clinic.

### Synergistic Bactericidal Action of PDA Nanosystem Mediated PTT

According to the role of the PDA nanosystem in antitumor therapy, it can also be applied for antibacterial therapy to reduce drug resistance. The high heat generated by the PDA nanosystem has been shown to be effective in removing bacterial biofilms (Borzenkov et al., 2020). In order to strengthen the antibacterial effects, antibiotic substance is often introduced into the PDA nanosystem, which allows the delivery of antibiotics for selective bacterial killing together with PTT (Tong et al., 2020; Xi et al., 2020; Sun et al., 2021; Xiao et al., 2021). For example, the drug-loaded PDA nanosystem can effectively kill bacteria near a wound and promote wound healing upon irradiation (Gao et al., 2019; Liang et al., 2019; Yin et al., 2020). Moreover, in recent years, Yuan’s team has shown that Gram-positive bacteria can be effectively killed by PTT, when combined with other antibacterial treatments, such as nitric oxide and carbon monoxide (Yuan et al., 2020; Yuan et al., 2021).

**FIGURE 2:**
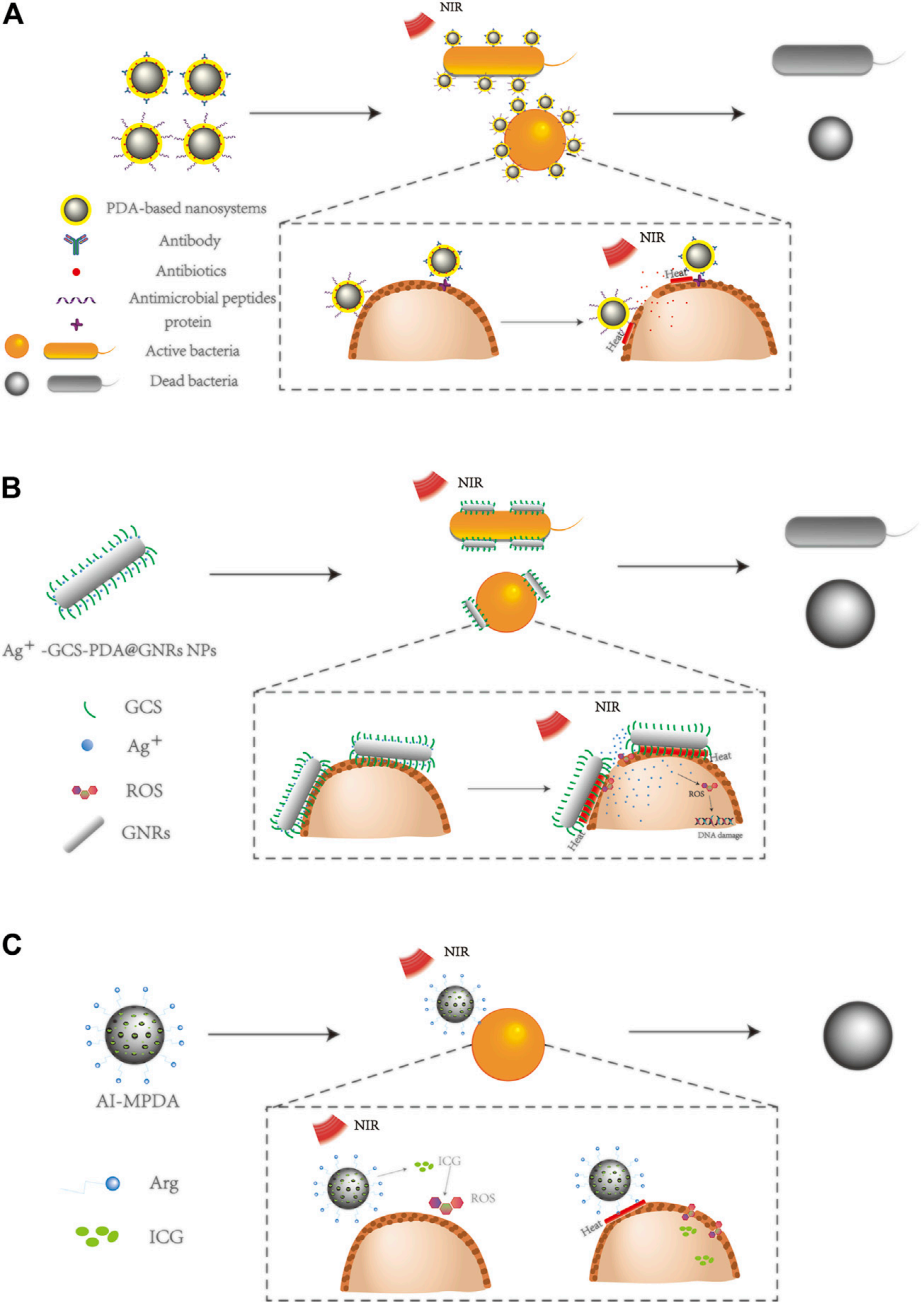
Different ways to enhance the antibacterial effect of the PDA nanosystem. **(A)** Specific antibody-enhanced targeting effects resulted in more effective bacterial killings in PDA nanosystem-mediated PTT. **(B)** Metal ions enhanced antibacterial effects in PDA nanosystem-mediated PTT; taking the PDA and glycol chitosan-coated gold nanorods (GCS-PDA@GNRs) as an example, the loading of silver ions into the nanosystem significantly enhanced the antibacterial efficiency (Liu et al., 2018). **(C)** ROS inductions to enhance the antibacterial effects of PDA nanosystem-mediated PTT, taking the phototherapeutic nanoplatform AI-MPDA composed of L-arginine (L-Arg), indocyanine green (ICG), and mesoporous PDA as an example (Yuan et al., 2020).

Up to now, how to use PTT-introduced high temperature to effectively remove bacterial biofilm with few effects on normal cells is the most important issue to be solved. Firstly, the most convenient and efficient method is to enhance the targeting effect of the PDA nanosystem against the cell membrane of different bacteria, which would result in the reduced accumulation of the PDA nanosystem surrounding the unexpected normal tissues or normal cells (Figure 2A). Some antibodies against bacterial surface proteins, such as anti-SPA (aSpa) antibody, anti-lipoprotein (aLpp) antibody, and anti-manganese transporter (aMntC) antibody, can be conjugated to the PDA nanosystem for specifically targeting Gram-positive *S. aureus* (Meeker et al., 2018).

For Gram-negative bacteria, the anti-outer member protein (aPa) antibody has been shown to target *Pseudomonas aeruginosa* (*P. aeruginosa*) for enhanced bacterial killings (Meeker et al., 2018). By conjugation with a specific antibody, the PDA nanosystem has been proved to show enhanced photothermal killing effects with increased drug delivery against bacteria, therefore resulting in much more potent antibacterial effects (Black et al., 2014; Meeker et al., 2018; Zhang Y. et al., 2019). Combination of the PDA nanosystem mediated PTT with active antibacterial components for more effective bacterial killings.

There are lots of active antibacterial components that can inhibit bacterial growth alone, which reminds us the use of the combination of these components with PTT for more effective bacterial killings. A substance with broad-spectrum antibacterial functions called antimicrobial peptide (AMP) has also been shown to preferentially bind to bacteria due to its conformation and charge properties (Yeaman and Yount, 2003). The combination of AMP with the PDA nanosystem can achieve synergistic antibacterial effects, which not only enhances its targeting ability but also increases its antibacterial ability due to the antibacterial effects of AMP (Fan XL. et al., 2019; Carratala et al., 2020). Through strengthening the targeted effects of the PDA nanosystem, the efficacy of antibiotics and the photothermal effect of nanoparticles have been proved to be significantly improved, which provides more strategies to decrease the dosage of antibiotics and reduce bacterial resistance.

Copper, silver, zinc, and other metal ions have been proved to affect the growth of bacteria and destroy the stability of the bacterial membrane structure at a certain concentration (Chernousova and Epple, 2013; Ye et al., 2020). Therefore, combining these metal ions with the PDA nanosystem would allow the antibacterial characteristics of metal ions to enhance the antibacterial effects of the PDA nanosystem. For example, Ag + -GCS-PDA@GNRs NPs have been found to release silver ions under acidic conditions or laser irradiation. Silver ions could damage cell membranes and further change the sensitivity of bacteria to heat. When bacteria are damaged by the temperature rise induced by PTT, more silver ions would enter into the bacteria cell and lead to DNA damage (Figure 2B). These synergistic effects could significantly improve the bactericidal efficiency of the PDA nanosystem (Liu et al., 2018). In addition, Ag + has also been proved to have a variety of functions that can induce the production of ROS to lead to bacterial destruction and enhance the antibacterial effect of Au (Xiao et al., 2021). Other metal ions also have similar antibacterial properties that have been found in other studies (Xu Q. et al., 2020; Ma et al., 2020; Yang et al., 2020), which shows the potential to enhance the antibacterial effects of PTT by combining the PDA nanosystem and functional metal ions.

Additionally, other materials can also be oxidized to produce ROS, which are capable of disrupting cell membrane structures through lipid oxidation and protein cross-linking, and can also cause DNA damage. The pathways initiated by ROS have been shown to be very effective in antibacterial treatment. It is well known that glutathione (GSH) plays very important roles in cell functions. Yuan et al. reported a functional molybdenum disulfide (MoS_2_)/PDA-arginine-glycine-aspartic acid (RGD) nanosystem coating on titanium (Ti) implant, which allowed accelerated GSH oxidation *via* photothermal energy and induced intrinsic ROS-independent oxidative stress damage deriving from MoS_2_ nanosheets (Yuan et al., 2019a). Hyperthermia induced by photothermal effects further accelerated the GSH consumption and ROS-independent oxidative stress to destroy the integrity of bacterial membranes, which resulted in effective clearance of *S. aureus* contamination (Yuan et al., 2019a).

Moreover, according to iron-catalyzed Fenton-type reaction that converts H_2_O_2_ into highly reactive hydroxyl, the introduction of transition metal ions into the PDA nanosystem could also produce greater toxic effects against bacteria (Tang et al., 2021). Xu et al. reported a kind of PDA nanosystem coating on hydroxyapatite (HAp) incorporated with gold nanoparticles (Au-HAp), which produced large amounts of hydroxyl radicals (OH) *via* catalysis of a small concentration of H_2_O_2_ to render bacteria more vulnerable to the temperature change (Xu et al., 2018). Moreover, by combining catalysis with near-infrared (NIR) PTT, Ti-Nd-PDA-Fc provides safe, rapid, and effective antibacterial activity compared to OH or PTT alone, thus accelerating wound healing more effectively (Song et al., 2020). Yuan et al. presented an all-in-one phototherapeutic nanoplatform consisting of l-arginine (l-Arg), indocyanine green (ICG), and PDA, to eliminate the already-formed biofilm more effectively by causing a cascade catalysis of l-Arg to release nitric oxide (NO) (Yuan et al., 2020). This NIR-irradiated PDA nanosystem not only prevented bacterial colonization but also realized a rapid recovery of infected wounds. In combination with other studies, the photothermal effects of the PDA nanosystem have been shown to be significantly enhanced by ROS production, which enhances oxidation and makes cell membranes more vulnerable to the destruction induced by PTT (Figure 2C) (Yuan et al., 2019b; Song et al., 2020; Su et al., 2021).

**FIGURE 3:**
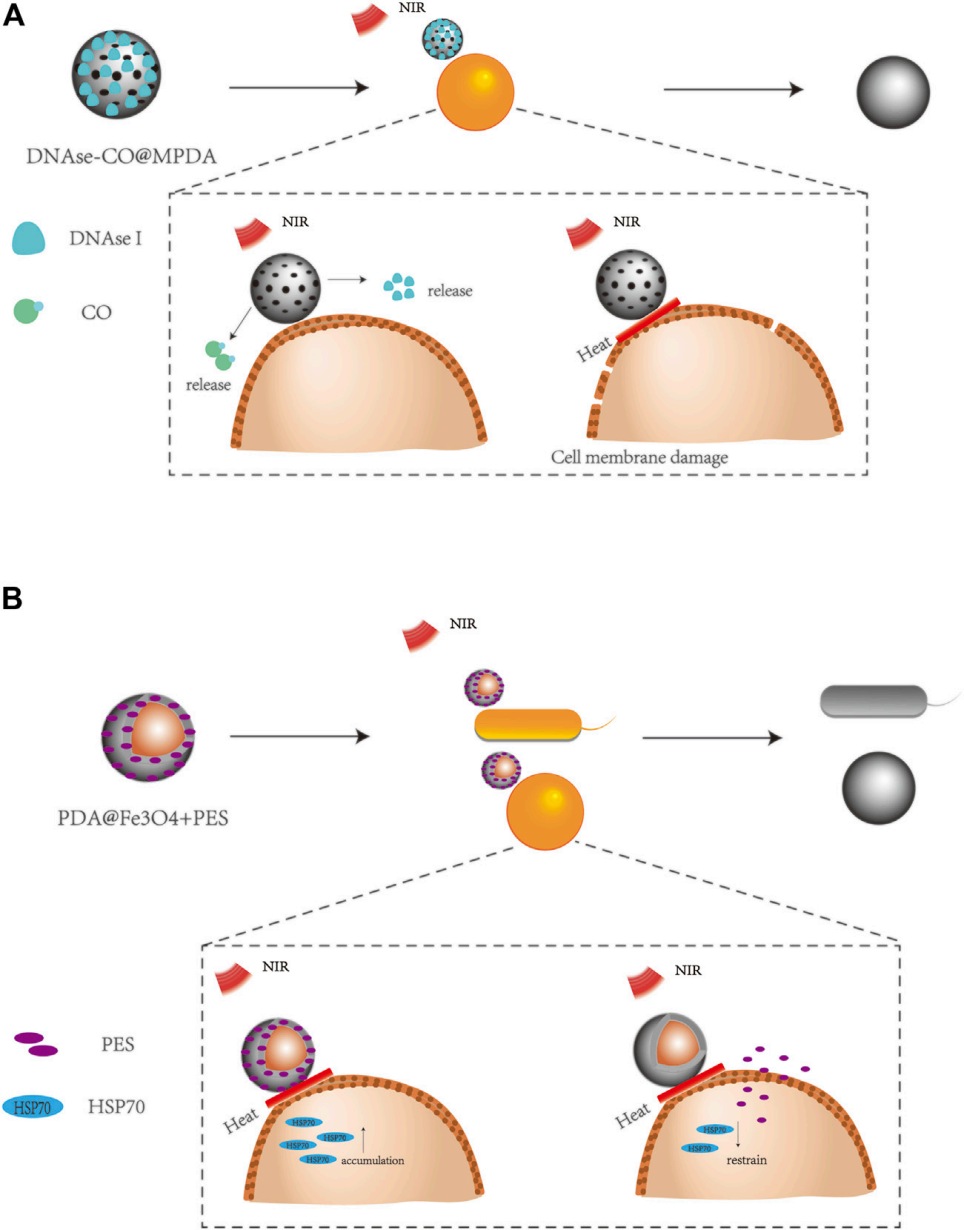
Different ways to enhance the antibacterial effect of the PDA nanosystem. **(A)** DNAse I can be used to destroy cell membrane densification for enhanced antibacterial effects, taking the thermosensitive CO gas releasing donor (FeCO)-encapsulated MPDA NPs with covalently fixed deoxyribonuclease I (DNase-CO @MPDA NPs) as an example (Yuan et al., 2021). **(B)** Inhibition of heat shock protein accumulation to enhance antibacterial effects, taking the PDA-encapsulated Fe_3_O_4_ and 2-phenylethyl sulfonamide (PDA@Fe_3_O_4_+PES) as an example (Liu et al., 2016).

Furthermore, taking the advantages of the synergistic antibacterial effects to achieve the bactericidal goal, some studies have aimed at the synthesis of the cell membrane and the protective mechanism of bacteria. For example, eDNA plays an important role in bacterial adhesion and synthesis of the cell membrane, which could serve as an antibacterial target. Yuan et al. combined deoxyribonuclease I (DNase I) with the PDA nanosystem to affect the synthesis and compactness of the bacterial biofilm, thus improving the efficiency of PTT (Figure 3A) (Yuan et al., 2021). The upregulation of heat shock proteins has been proved to provide protection for cells to gain tolerance against stress, such as PTT-induced hyperthermia. Therefore, 2-phenylacetylene sulfonamide (PES) and the PDA-based nanosystem were combined to inhibit heat shock proteins to reduce the heat resistance of bacteria, thus improving the photothermal inactivation of bacteria (Figure 3B) (Liu et al., 2016).

Due to the extensive use of antibiotics, many bacteria have developed drug resistance that seriously threatens human life. Considering the convenience to avoid drug resistance, physical antibacterial methods are attracting more and more attention (Pang et al., 2019; Sun et al., 2020). As a kind of physical bactericidal strategy, PTT based on PDA nanomaterials has also been proved to significantly destroy the cell membrane of methicillin-resistant *S. aureus* (MRSA). For example, PDA/Cu CS hydrogel was found to affect the permeability of the bacterial membrane by their bacteriostatic and thermal effects of Cu2+, which also interfered with the replication of RNA and DNA by producing ROS to produce obvious genotoxicity against bacteria (Xu Q. et al., 2020). DNase I has also been shown to have the ability to destroy bacterial cell membranes; thus, DNase-CO@MPDA NPs also indicated a powerful killing effect on MRSA by combining the antibacterial effects of DNase, CO, and PDA-mediated PTT (Yuan et al., 2021). Similar functional nanoplatforms could also be used for the treatment of Gram-negative bacteria with multiple-drug resistance (Zhao et al., 2020). This shows that the PDA nanosystem has great potential to explore new antibacterial modes to reduce the occurrence of multiple-drug-resistant bacteria.

Some studies have also verified the *in vivo* antibacterial effects of the mPDA nanosystem using wound models. In addition to the thermal effect brought by the PDA nanosystem, combined with drug delivery, release of metal ions, oxidative stress, and so on, it showed excellent antibacterial effect and wound healing ability. The combined use of these antibacterial mechanisms showed significantly better effects than the use of antibiotics alone and phototherapy (Liu et al., 2018; Xu et al., 2018; Ma et al., 2020). Cu-GA-CA-PDANRs showed the best effects in these studies, leading to almost complete wound healing within 9 days (Sun et al., 2021). For MRSA, PDA/Cu-Cs hydrogel and DNAse-CO@MPDA also showed excellent bactericidal efficiency and significantly reduced wound injury within 14 days (Xu Q. et al., 2020; Yuan et al., 2021). All these prove that the antibacterial function mediated by the PDA nanosystem has great application potential.

In summary, PTT mediated by the PDA nanosystem needs to be enhanced by adjusting the shape, size, irradiation wavelength, and irradiation area of nanomaterials in the single antibacterial treatment, and the effect of change is often unsatisfactory. However, on this basis, the PDA nanosystem can be synergistic with antibodies, metal ions, oxides, and enzymes to improve the effect of PTT. In Table 1, we summarize some typical examples of the photothermal effects of different approaches to enhance PDA nanosystems, which will help us understand how to synergically enhance antibacterial PTT through PDA nanosystems.

**TABLE 1:** Typical study on antibacterial activity of the PDA nanosystem.

Nanomaterial	Mechanism	Laser	Wavelength	Bacteria	References
Ag^+^-GCS-PDA@GNRs	Photothermal effect, metal ion release	NIR	808 nm	*S. aureus*	Liu et al. (2018)
				*E. coli*	
Cu-GA-CA-PDANRs	Drug delivery, photothermal effect	NIR	808 nm	*S. aureus*	Sun et al. (2021)
				*E. coli*	
CHX@CP3	Drug delivery, photothermal effect	NIR	808 nm	*S. aureus*	Tong et al. (2020)
PPCP matrix	Drug delivery, photothermal effect	NIR	808 nm	*S. aureus*	Xi et al. (2020)
Van@ZIF-8@PDA	Drug delivery, photothermal effect	NIR	808 nm	*S. aureus*	Xiao et al. (2021)
				*E. coli*	
PDA NP-Cip/GC hydrogel	Drug delivery, photothermal effect	NIR	808 nm	*S. Aureus*	Gao et al. (2019)
				*M. luteus*	
				*E. coli*	
				*P. vulgaris*	
GT-DA/chitosan/CNT hydrogels	Drug delivery, photothermal effect	NIR	808 nm	*S. aureus*	Liang et al. (2019)
				*E. coli*	
SP@MX-TOB/GelMA	Drug delivery, photothermal effect	NIR	808 nm	*S. aureus*	Yin et al. (2020)
				*E. coli*	
DNAse-CO@MPDA	DNase active, photothermal effect, CO release	NIR	808 nm	MRSA	Yuan et al. (2021)
AI-MPDA	ROS generation, NO release, photothermal effect	NIR	808 nm	*S. aureus*	Yuan et al. (2020)
AuNC@PDA	Antigen antibody reaction to target cell membranes, photothermal effect, drug delivery	NIR	808 nm	*S. aureus*	Meeker et al. (2018)
				*P. aeruginosa*	
Ab-Au@Ag-NRs	Antigen antibody reaction to target cell membranes, photothermal effect, metal ion release	NIR	480–850 nm	*S. epidermidis*	Black et al. (2014)
				*E. coli*	
MoS_2_@PDA-PEG/IgG NSs	Antigen antibody reaction to target cell membranes, photothermal effect	NIR	785 nm	*S. aureus*	Zhang et al. (2019c)
MagI-PEG@PDA NP	Antibacterial peptide targeting bacterial effect, photothermal effect	NIR	808 nm	*E. coli*	Fan et al. (2019b)
PDA@Van-Ag	Photothermal effect, metal ion release, drug delivery	NIR	808 nm	*S. aureus*	Ma et al. (2020)
				*E. coli*	
PDA/Cu-CS hydrogel	Photothermal effect, metal ion release	NIR	808 nm	MRSA	Xu et al. (2020b)
				*E. coli*	
GNR–PDA@Zn	Photothermal effect, metal ion release	NIR	808 nm	*S. aureus*	Yang et al. (2020)
				*E. coli*	
MoS_2_/PDA-RGD	Oxidative stress, photothermal effect	NIR	808 nm	*S. aureus*	Yuan et al. (2019a)
				*E. coli*	
PDA@Au-HAp	Oxidative stress, photothermal effect	NIR	808 nm	*S. aureus*	Xu et al. (2018)
				*E. coli*	
Ti-Nd-PDAFc	ROS generation, photothermal effect	NIR	808 nm	*S. aureus*	Song et al. (2020)
				*E. coli*	
Ti-M/I/RGD	ROS generation, photothermal effect	NIR	808 nm	*S. aureus*	Yuan et al. (2019b)
PDA-Cur	ROS generation, photothermal effect	NIR	808 nm	*S. aureus*	Su et al. (2021)
				*E. coli*	
PDA@Fe_3_O_4_+PES	photothermal effect, Inhibition of heat shock protein inhibitors	NIR	785 nm	*S. aureus*	Liu et al. (2016)
				*E. coli*	

### Potentials of PDA Nanosystem Mediated PTT for Intracellular Bacteria Clearance

It is worth noting that the current research about PDA nanosystem-mediated PTT is still focusing on the direct killing effects against bacteria, which ignore their potential uses for intracellular bacterial pathogen clearance. Some important intracellular bacterial pathogens, such as *M. tuberculosis* (Mtb) and *Brucella*, are still deadly threatening human life worldwide. Here, we set Mtb as an example of stubborn intracellular bacteria to discuss the potential of PTT in intracellular bacterial clearance. As one of the most successful pathogens in human history, Mtb has developed multiple innate and adaptive immunological escape mechanisms to avoid the host killing effects (Zhai et al., 2019), which resulted in the hiding of Mtb in the host cells to develop latent or active tuberculosis (TB). In the case of Mtb therapy, the low efficiency of antibiotics leads to the current standard treatment of Mtb, which is a regimen of frontline combination chemotherapy with multiple antibiotics for at least 6 months. These anti-Mtb treatments have indicated lots of limitations, such as excessive treatment duration, strong drug toxicity, and increasing risk of resistant mutants. Therefore, there is an urgent need of novel strategies for more effective TB treatments and drug resistance restrictions.

Currently, host-directed therapy (HDT) is expected to fight intracellular bacterial infections and improve the efficiency of TB treatment by regulating host cell immunity (Paik et al., 2019). As one of the most important intracellular degradation systems that deliver cytoplasmic constituents to the lysosome for degradation, autophagy has been proved to be a critical innate immunological response against Mtb (Paik et al., 2019). However, Mtb can inhibit the autophagy of host cells, which is also an immunological escape mechanism of Mtb (Zhai et al., 2019). Targeting autophagy has been proposed as an attractive strategy for intracellular Mtb clearance, which has been proved by bazedoxifene (BZA)-promoted autophagy to inhibit intracellular Mtb (Ouyang et al., 2020).

It has been widely known that the insufficient heat damage induced by PTT cannot completely eradicate tumor due to the existence of multiple intracellular defense mechanisms in cancer cells, especially autophagy, which can be activated to avert and repair cellular damage caused by PTT-induced heating (Zhang X. et al., 2019). This interesting mechanism reminds us that the host cells of Mtb might also have some active response against the insufficient heat damage induced by PTT, which therefore might promote the autophagy of Mtb-infected host cells for more effective Mtb clearance. In the study of using Au-Ag@PDA NP-mediated PTT for tumor treatment, PTT was found to induce the formation of autolysosomes and produce ROS upon thermal effect (Zhao et al., 2018). These results collectively suggested the use of PDA nanosystem-based PTT to activate host cell autophagy for the intracellular Mtb killings.

Additionally, Mtb can also evade host cell immunity by inhibiting the host cell apoptosis (Zhai et al., 2019). When using the PDA nanosystem to treat tumors, it can increase the expression of Bax and reduce the expression of Bcl-2, resulting in potential damage to the mitochondrial membrane, and release activated cytochrome c to activate caspase-3/caspase-8 apoptosis (Zhao et al., 2018). Thus, there would be another interesting hypothesis that PDA nanosystem-mediated PTT might also be used for antimicrobial therapy by regulating host cell apoptosis.

Another attractive property of PTT is that the mild PTT treatment can also regulate innate immunity for potential immunotherapy (Li J. et al., 2021; Miller et al., 2021). PDA nanosystems have been found to promote dendritic cell maturation and activate CD8^+^ T lymphocytes (Yan S. et al., 2019; Wang D. et al., 2020). Perforin and granzyme secreted by activation of CD8^+^ T lymphocytes have been reported to be significantly associated with the inhibition of intracellular Mtb growth (Yang R. et al., 2021). Therefore, CD8+T lymphocytes might also be activated by PDA nanosystem-mediated PTT to promote the release of perforin and granzyme, which needs to be further investigated. Although the escape mechanisms of Mtb are not fully understood, it would be an interesting topic to explore the regulation of autophagy, apoptosis, and innate immunity in host cells upon PDA nanosystem-mediated PTT and further explore whether these effects are helpful for intracellular Mtb clearances or not. This will further expand the application range of the PDA nanosystem to improve the therapeutic function of bacterial infection and reduce the occurrence of multiple-drug-resistant bacteria.

More importantly, for the multidrug-resistant Mycobacteria, the acquired drug resistance rate of mycobacteria is also increasing with the use of antibiotics. We know that the mechanisms of *mycobacterium* drug resistance can be roughly divided into (1) the barrier mechanism, that is, the change of permeability of the outer membrane of the cell which interferes with the drug transport process; (2) drug degradation or inactivation; and (3) drug target or enzyme activity-related gene mutation-induced drug failure (Khawbung et al., 2021). The bioactivity and good drug delivery performance of PDA nanosystems can reduce the degradation rate of drugs *in vivo* and target the host cells to regulate their function and reduce the amount of drugs for a better killing effect, which therefore reduce the generation of acquired drug resistance of Mtb(Singh et al., 2020). Currently antibacterial drugs are being developed to treat resistance caused by genetic mutations (Kaur et al., 2021). For example, the FNDR-20081, PDA nanosystem can be used as a good biological carrier to combine FNDR-20081 with first-line and second-line anti-tuberculosis drugs to produce better killing effects. Due to limited research on the mechanisms of drug resistance of MDR, the potential of PDA nanosystems to reduce the generation of MDR thus requires more precise mechanism studies.

## Conclusion and Perspectives

PDA has shown great potential to act as a powerful photothermal agent, which is not only due to its high photothermal efficiency but also due to its strong adhesion properties to form films on most solid materials. Thus, PDA nanomaterials, including PDA nanomaterials and PDA-coated nanomaterials, have indicated some very interesting properties, such as high chemical reactivity, good metal coordination ability, excellent biocompatibility and quenching effect, and outstanding thermal conversion ability. The PDA nanosystem can introduce hyperthermia into tumors through PTT and can be combined with other strategies such as inhibition of autophagy, induction of apoptosis, and immune regulation to achieve synergistic enhancement of antitumor efficiency. Immunotherapy may be a promising approach for future applications of PDA materials, for example, by loading appropriate immunological regulators into the PDA nanosystem that can activate antitumor immune cells for more effective cancer cell killings.

Here, we further discussed the potential of PDA nanosystem-mediated PTT for antibacterial treatment. PTT can kill bacteria by laser irradiation-induced heat due to the excellent photothermal efficiency of the PDA nanosystem; however, PTT-induced hyperthermia can also introduce strong side effects on normal cells. How to trigger bacterial death without affecting the metabolism status of normal cells by the PDA nanosystem remains to be further investigated.

We also introduced some strategies to improve the efficacy of PTT in the current PDA nanosystem for antibacterial research. The increased targeting effects of the PDA nanosystem against bacteria could further enhance their selectivity to achieve enhanced antibacterial efficiency, also by adding some functional substances, such as metal ions with different antibacterial effects for collaborative treatment. The addition of antibacterial metal ions into the PDA nanosystem could not only reduce the temperature requirements for effective PTT treatment but also greatly improve the safety and efficiency of PTT. The direct regulation of ROS has been widely used to enhance the efficiency of PDA nanosystem-mediated PTT, which could serve as a very useful strategy to develop more effective PTT treatments. Moreover, similar with some other nanomaterial-mediated PTT, the direct regulation of heat shock protein expression could also contribute to the efficiency of PDA nanosystem-mediated PTT. These strategies could serve as potential arsenal to further enhance the PTT treatments based on the PDA nanosystem and finally contribute to antibacterial treatments.

In order to accelerate the development of PTT in bactericidal and future clinical application, there are still many obstacles and problems to be solved. As for nanomaterials, first of all, the surface charge, morphology, and crystal structure of different nanomaterials determine their antibacterial activity, and the manufacturing process of nanomaterials is complicated. How to economically and efficiently produce a PDA nanosystem with good antibacterial activity and safety is currently urgent to be explored. Secondly, the current national laser use standard of the United States is 0.33 W/cm^2^ of 808 nm, which is very strong irradiation for skin exposure. Slightly deeper tissue infections using such high-power density would expose normal cells to the risk of hypothermia injury, which therefore requires more research works to explore the ultra-low dose of laser wavelengths and areas to avoid damage and achieve better therapeutic effects. Finally, PDA has very good biocompatibility and low toxicity, but information on the long-term toxicity of the PDA nanosystem against human remains unclear. Moreover, except for the potential toxicity of the PDA nanosystem itself, whether there is potential ingredient production during the metabolism of the PDA nanosystem would also be a critical issue to be concerned.

For future antibacterial studies of PDA-mediated PTT, firstly, we need to explore a more effective combination mode for extracellular bacterial treatment to achieve better therapeutic effects. Secondly, in addition to further exploring the escape mechanism of intracellular bacteria, we could also use some well-known mechanisms to develop targeted host cell therapies. We believe that these contributions would further benefit the current therapies against different kinds of bacteria.

Drug resistance has become one of the most urgent global issues due to the lack of effective antibiotics to avoid the emergence of resistant mutants, which urges us to explore more powerful strategies against the drug-resistant mutants. PTT has been proved to be an effective method to kill bacteria directly even without drug treatments, and it can also be applied together with some antibiotics for more effective bacterial killings. Taking the advantages of their high biocompatibility and potent photothermal efficiency, PDA-based nanosystems are expected to not only provide novel possibilities, against the drug resistant mutants, but also avoid the emergence of drug resistance.

Taking the advantages into account, we found that PDA nanosystem-mediated PTT has shown a very good prospect for clinical application due to its low toxicity, high biological activity, and strong ability for drug encapsulation. Secondly, PDA can not only be used to make hydrogel materials for skin surface wounds to kill bacteria and promote healing but also be designed to penetrate into bone tissue to avoid bacterial infection of bone tissue after surgery. The third advantage of the PDA nanosystem is its wide range of actions except for the thermal effect alone, which can be combined with metal ions, oxidative stress, drug delivery, antibody targeting, and so on, to significantly enhance the antibacterial functions. Last but not least, the PDA nanosystem can reduce the use of antibiotics and increase drug utilization to slow or reduce the formation of multidrug-resistant bacteria. However, for successful clinical application, there are still lots of critical issues that need to be overcome, for example, how to kill bacteria without affecting the condition of normal cells to avoid normal tissue damage, how to apply it for intracellular bacterial killings effectively, and how to use it for the treatment of patients with drug-resistant mutant infections.

Thus, although PTT provides a promising approach against pathogens, there are still many obstacles that affect its clinical use. More experiments and observation are needed to evaluate the performance and safety of the PDA nanosystem for antibacterial PTT treatment. Moreover, we believe that as one of the most functional nanomaterials with strong biocompatibility, the PDA nanosystem would play more and more important roles for the development of antibacterial PTT strategies.
